# Mesoporous Calcium-Silicate Nanoparticles Loaded with Low-Dose Triton-100+Ag^+^ to Achieve Both Enhanced Antibacterial Properties and Low Cytotoxicity for Dentin Disinfection of Human Teeth

**DOI:** 10.3390/pharmaceutics13091518

**Published:** 2021-09-19

**Authors:** Mengting Duan, Wei Fan, Bing Fan

**Affiliations:** The State Key Laboratory Breeding Base of Basic Science of Stomatology (Hubei-MOST) and Key Laboratory of Oral Biomedicine Ministry of Education, School and Hospital of Stomatology, Wuhan University, Wuhan 430000, China; mengtingduan01@gmail.com

**Keywords:** mesoporous, nanoparticle, *Enterococcus faecalis*, silver, Triton X-100, root canal disinfection

## Abstract

Mesoporous calcium-silicate nanoparticles (MCSNs) are excellent biomaterials for controlled drug delivery and mineralization induction. In this study, MCSNs were loaded with low-dose silver ion (Ag^+^) and Triton X-100 (TX-100) as the M-AgTX to achieve both enhanced antibacterial properties and low cytotoxicity for dentin disinfection. The physicochemical property, biocompatibility, infiltration ability into dentinal tubules, anti-bacterial ability against both planktonic *Enterococcus* *faecalis* (*E. faecalis*) and its biofilm on dentin, effects on dentin microhardness and in vitro mineralization property were systematically investigated. Results confirmed that the MCSNs and M-AgTX nanoparticles showed typical morphology of mesoporous materials and exhibited sustained release of chemicals with an alkaline pH value over time. M-AgTX also exhibited excellent biocompatibility on MC3T3-E1 cells and could eliminate 100% planktonic *E. faecalis* after 48-h treatment. On dentin slices, it could enter dentinal tubules by ultrasonic activation and inhibit the growth of *E. faecalis* on dentin. M-AgTX could completely inactive 28-day *E. faecalis* biofilm. TEM confirmed the destruction of cell membrane integrity and Ag^+^ infiltration into bacteria by M-AgTX. Besides, dentin slices medicated with M-AgTX nanoparticles displayed an increased microhardness. After being immersed in SBF for 7 days, apatite crystals could be observed on the surface of the material tablets. M-AgTX could be developed into a new multifunctional intra-canal medication or bone defect filling material for infected bone defects due to its sustained release profile, low cytotoxicity, infiltration ability, enhanced anti-bacterial and mineralization features.

## 1. Introduction

*Enterococcus faecalis* (*E. faecalis*) is a Gram-positive facultative anaerobic bacterium reported to be resistant to many anti-bacterial medications or agents, that can cause refractory infection and reinfection of the root canal system of human teeth [1,2]. Its ability to grow into dentinal tubules and other root canal irregularities make it very difficult to eliminate from the root canal even with meticulous canal disinfection management practices [2,3]. Therefore, the search for more effective antibacterial agents against *E. faecalis* has always been a research focus of root canal disinfection studies [4].

Calcium hydroxide (Ca(OH)_2_) is the most popular intracanal disinfection medicament so far due to its high alkaline pH due to the hydroxyl ions it releases [5]. However, it is unable to eliminate *E. faecalis* as *E. faecalis* shows strong resistance to alkalinity, and the prolonged treatment with Ca(OH)_2_ also could result in reduced flexural strength and fracture resistance of dentin, thus increasing the risk of tooth fractures [6,7,8]. Chlorhexidine (CHX) is thought as one of the “golden standards” for evaluating the anti-bacterial ability of intracanal medicaments due to its strong and broad-spectrum anti-bacterial ability. Despite this, its high cytotoxicity, acidic pH, potential discoloration effects and toxic precipitation when co-used with sodium hypochlorite (NaClO, an often used root canal disinfecting irrigant) severely limit its application [4,9,10]. In addition, the substantivity of antibacterial chemicals on dentin surface is deemed as an important property to achieve a prolonged anti-bacterial effect. What’s more, the root canal infection is always associated with the loss of peri-apical alveolar bone tissues, hence pro-osteogenic property is also another important feature for ideal intracanal medicaments [11].

Mesoporous calcium-silicate nanoparticles (MCSNs) have been reported as an ideal carrier for various bioactive molecules delivery due to their special porous structures and excellent biocompatibility [12,13,14,15,16]. MCSNs have been widely used to deliver antibiotics, anti-cancer drugs, and other chemicals [16,17]. Besides, it has also been reported that MCSNs could be driven into dentinal tubules by ultrasound activation [15]. Its continuous calcium (Ca) and silicate (Si) ions release properties could promote mineralization and osteogenic differentiation of stem cells in vitro, which enable the MCSNs to be a promising filling material candidate for bone defects [13,15,18]. Despite this, the anti-bacterial activity of MCSNs is limited [13,15].

As antibiotic resistance has become a global issue, metal ions showing antibacterial properties have become alternatives to antibiotics in many disinfection areas [19,20]. Silver ion (Ag^+^) is a strong broad-spectrum anti-bacterial agent and has been used for disinfection for centuries [21]. However, the irreversible pigmentation, high cytotoxicity, and especially the Ag^+^-resistance developed by many bacteria after prolonged exposure restrict its clinical applications [19,21,22,23]. One possible way to solve these problems is to co-use Ag^+^ with other materials, such as CHX, zinc ions, and simvastatin [19,24,25]. Triton X-100 (TX-100) as a nonionic detergent, is an essential tool for the study of biological membrane because it could incorporate with liposomes of cell membranes, thus increasing the penetrability of membrane [26]. According to our previous study, TX-100 was confirmed to help the infiltration of Ag^+^ into bacteria cells, thus show synergistic enhanced anti-bacterial effect even at very low concentrations.

To test the hypothesis, the aim of this study was to synthesize a new MCSNs containing low dose Ag^+^ and TX-100 as the M-AgTX, and its physicochemical features, releasing profile, biocompatibility, infiltration ability, anti-bacterial effect against *E. faecalis* on dentin, as well as the in vitro mineralization were systematically studied.

## 2. Materials and Methods

### 2.1. Materials

Cetyltrimethylammonium bromide (CTAB), Ca(OH)_2_ were purchased from Sigma-Aldrich Co. (St. Louis, MO, USA). Tetraethyl orthosilicate (TEOS) and methyl cellulose were obtained from Aladdin Industrial Corporation (Shanghai, China). Ammonium hydroxide, calcium nitrate tetrahydrate, silver nitrate (AgNO_3_), NaClO, ethylenediaminetetraacetic acid (EDTA) were obtained from Sinopharm Chemical Reagent Co., Ltd. (Shanghai, China). TX-100 was obtained from BioFroxx (Einhausen, Germany). Tris-HCL (1M, pH 7.4) was purchased from Aspen Biological (Wuhan, China). Brain heart infusion (BHI) were purchased from Becton-Dickinson (Franklin Lakes, NJ, USA). A Cell Counting Kit-8 (CCK-8) was obtained from Dojindo Laboratories (Kumamoto, Japan).

### 2.2. Synthesis of MCSNs and M-AgTX

The MCSNs were synthesized based on our previously study [13]. Briefly, CTAB (6.6 g) was dissolved in 600 mL of double-distilled water (ddH_2_O), followed by dropwise addition of ammonium hydroxide (12 mL). After stirring for 30 min, TEOS (30 mL) and calcium nitrate tetrahydrate (31.21 g) were added. After vigorous stirring for 3 h, the products were washed three times with ddH_2_O and absolute ethanol, respectively. At last, the collected powders were dried at 60 °C in the oven overnight and calcined at 550 °C in the stove, referred to as MCSNs. Next 1% AgNO_3_ aqueous solution and 1% TX-100 were incorporated into MCSNs by 1-h vigorous stirring at 60 °C in the dark. After filtration, the powders were washed with absolute ethanol and dried overnight, referred to as M-AgTX.

### 2.3. Characterization of MCSNs and M-AgTX

The ultrastructural features of MCSNs and M-AgTX were observed by field emission scanning electron microscopy (FE-SEM; Sigma, Zeiss, Baden-Württemberg, Germany) and transmission electron microscopy (TEM; JEM-2100, JEOL, Tokyo, Japan). The elements of the particles were examined by energy dispersive spectrometry (EDS; Quanta 200, Thermo Fisher Scientific, Waltham, MA, USA). The specific surface area, pore volume, and pore size distribution according to nitrogen-adsorption isotherms (ASAP 2020, Micromeritics, Norcross, GA, USA) were measured using Brunauer-Emmett-Teller as well as the Barrett-Joyner-Halenda analyse. Fourier transformed infrared spectroscopy (FTIR; Nicolet5700, Thermo Fisher Scientific) was used to evaluate the combination between MCSNs and TX-100 in M-AgTX.

### 2.4. pH Measurement and Release Profile

The pH change was measured by soaking 100 mg MCSNs or M-AgTX in 20 mL of ddH_2_O at 37 °C in the darkness. At 1, 3, 6, 9 days, a pH meter (Sartorius AG, Goettingen, Germany) was used to measure pH value. For detecting the release of Ca^2+^, SiO_3_^2−^ and Ag^+^, 20 mg MCSNs or M-AgTX were soaked in 10 mL of Tris-HCL at 37 °C in the darkness. For detecting the release of TX-100, 100 mg M-AgTX were soaked in 2 mL of ddH_2_O under the same condition. At the same time interval as above, half of the mixture was removed to measure the ions or TX-100 concentration, meanwhile fresh identical solutions were added. The concentrations of Ca^2+^, Ag^+^, SiO_3_^2−^ were measured by inductively coupled plasma (ICP-AES; IRIS Intrepid II XSP, Thermo Fisher Scientific, Waltham, MA, USA) and the concentration of TX-100 was measured by High Performance Liquid Chromatography (HPLC; Agilent 1100 system, Agilent Technologies, Santa Clara, CA, USA), respectively. Testing was performed in triplicate.

### 2.5. Cytotoxicity Test

Firstly, 100 mg of nanoparticles were soaked in 10 mL of α-minimum essential medium (α-MEM) with 1% penicillin/streptomycin at 37 °C for 24 h. After sterilization through a 0.22 μm filter unit (Merck Millipore Ltd., Darmstadt, Germany), the supernatant was collected and mixed with 10% fetal bovine serum. Dilutions of 1, 2, 5 mg/mL were used. Then 100 μL of MC3T3-E1 (ATCC) (10 × 10^3^ cells/well) were seeded into each well of a 96-well plate. Each group included 6 repeated wells. After 24-h incubation at 37 °C with 5% CO_2_, 200 μL fresh α-MEM and 10 μL extracts were added to replace the medium. After incubation for 2, 4, 6 days, the medium was removed, cells were washed with phosphate-buffered saline (PBS) and then cultured with 110 μL pre-mixed solution containing 100 μL α-MEM and 10 μL CCK-8 at 37 °C for 2 h. Then the absorbance at 450 nm was measured by a micro-plate reader (Power Wave XS2, BioTek Instruments, Winooski, VT, USA). The group that cells cultured without extracts was used as the blank control, and 2% CHX treated group was used as the positive control. All the operations were carried out in the darkness.

### 2.6. Minimum Inhibitory Concentration (MIC) and Bactericidal Concentration (MBC) of MCSNs and M-AgTX

MIC and MBC were determined by a serial microdilution assay [13,27]. The nanoparticles extracts were prepared by soaking in BHI at 37 °C for 24 h, from starting solutions of 128 mg/mL, a serial of two-fold dilutions were made. Then 50 μL dilution was incubated with 50 μL *E. faecalis* (ATCC 29212) suspension (2 × 10^5^ CFUs/mL) into wells of a 96-well plate at 37 °C in the dark anaerobically (with 5% CO_2_ and 1% O_2_) for 24 h. An optical density measurement was conducted at 600 nm by a micro-plate reader. When the wells showed no turbidity compared with the blank control, the lowest concentration was defined as MIC. And after inoculating solutions from wells without turbidity onto BHI agar plates, the concentration which wells with the number of viable bacteria less than 4% of original bacteria was determined as MBC_96_. The test was repeated three times.

### 2.7. Anti-Bacterial Effect against Planktonic E. faecalis

A 1 mL suspension (1 × 10^8^ CFUs/mL) of *E. faecalis* was incubated anaerobically with 1, 2 and 5 mg M-AgTX or 5 mg of MCSNs nanoparticles at 37 °C in the dark. Original *E. faecalis* was used as the blank control. At 12, 24, 36, 48-h time intervals, 10 μL of the solution was removed to inoculate a BHI agar plate for another 24 h, then CFUs were counted, and the total number of bacteria in 1 mL of mixture was calculated. Considering that the amount of *E. faecalis* in MCSNs and blank control groups was difficult to count, the suspensions were diluted 10^6^-fold before inoculation. The test was repeated six times. The survival rate (%) was calculated using the following Equation (1):

(1) $$\mathrm{Survival}\;\mathrm{rate}\;(\%)=\frac{{{\mathrm{the}\;\log}_{10}}^{\;\mathrm{CFUs}\;\mathrm{of}\;\mathrm{treatment}\;\mathrm{group}\;}}{{{\mathrm{the}\;\log}_{10}}^{\;\mathrm{CFUs}\;\mathrm{of}\;\mathrm{blank}\;\mathrm{control}\;\mathrm{group}\;}}\times 100$$

where the value of log_10_^0^ not in the equation was defined as 0 in this study.

### 2.8. Dentinal Tubule Infiltration Ability and Anti-Bacterial Effect on Dentin Pretreated with M-AgTX

Extracted non-carious human third molars were collected with the approval of the Ethics Committee of the School and Hospital of Stomatology of Wuhan University. In brief, the dentin slices were firstly cut to a size of 4 mm × 4 mm × 1 mm (width × length × thickness), and after being polished, the slices were cleaned by successive ultrasonic washing in ddH_2_O, 5.25% NaClO and 17% EDTA for 4 min and in ddH_2_O for one more minute. Before use, all slices were autoclaved. To investigate the infiltration ability of nanoparticles into dentinal tubules, dentin slices were placed vertically in 0.5 mL ddH_2_O containing 5 mg of M-AgTX nanoparticles, and each side of the slices was subjected to passive ultrasonic vibration (Neutron P5 piezoelectric, courtesy of Acteon Satelec, Merignac Cedex, France) at scale 4 for 60 s. The slices were dried at 60 °C in the oven overnight before using FE-SEM to observe the adhesion of nanoparticles on the dentin surface. And one more slice was split with a hammer and chisel to expose the axial cross section of dentin tubules before FE-SEM observation.

For anti-bacterial effect tests on dentin pretreated with M-AgTX, dentin slices were pretreated with MCSNs, M-AgTX, or no particles as above. Each group included six slices. Then the slices were soaked in 100 μL suspension (1 × 10^4^ CFUs/mL) of *E. faecalis* at 37 °C anaerobically in the darkness for 7 days. After being washed with PBS, the slices were soaked in 6 mL fresh BHI, at 2, 4, 6, 8-h interval, 1 mL mixture was extracted and a spectrometer was used to measure the OD value at 600 nm. Meanwhile, one more slice in each group were desiccated, and the morphology and colonization of *E. faecalis* on the dentin surface were observed by FE-SEM.

### 2.9. Anti-Biofilm Activities of M-AgTX against E. faecalis Mature Biofilm on Dentin

Dentin slices were prepared as above. After autoclaving, they were placed in 3 mL of *E. faecalis* suspension (1 × 10^8^ CFUs/mL) and cultured anaerobically at 37 °C in the dark for 28 days. The BHI medium was refreshed every second day to ensure bacterial viability. MCSNs and M-AgTX pastes (mixed with ddH_2_O = 1:4) were prepared, Ca(OH)_2_ paste (mixed with ddH_2_O = 1:2) was used as a conventional intracanal medication. For positive control group, methyl cellulose was added into 2% CHX solution to form a gel, and for blank control group, methyl cellulose was added into the PBS solution. Each group included six slices. Dentin slices were embedded in paste or gel were incubated at 37 °C under humid conditions in the darkness for 7 days to stimulating the clinical root canal medication. After being washed by PBS, the slices were soaked in 10 mL BHI, at 2, 4, 6, 8, 10, 12, 24, 36-h interval, 1 mL suspension was taken out for OD value measurement as above. To observe the morphology, colonization and adhesion of bacteria on dentin directly, FE-SEM was conducted on one more slice.

### 2.10. Endocytosis of Nanoparticles by E. faecalis

In brief, 10 mL *E. faecalis* suspension (1 × 10^9^ CFUs/mL) was co-cultured with 5 mg of M-AgTX. After 24-h incubation, the M-AgTX-treated bacteria was obtained by centrifugation at 1000 rpm for 5 min. Then the bacteria were prepared by fixation, staining, dehydration, infiltration with polymer resin, oven-curing, and slicing via ultra-microtome (LEICA EM UC7, Leica, Wetzlar, Germany) before the TEM observation for endocytosis of M-AgTX by *E. faecalis*.

### 2.11. Microhardness Test

Dentin disks with a thickness of 1 mm were prepared from extracted wisdom teeth, and all dentin disks were cleaned as above. Six original dentin disks from each group were subjected to a digital microhardness tester (HXD-100TMC/LCD, Shanghai Taiming Optical Instrument Co,. Ltd., Shanghai, China) under a load of 50 g for 10 s, and the average microhardness of six random spots was calculated [15]. MCSNs, M-AgTX and Ca(OH)_2_ pastes, 2% CHX and PBS gels were prepared as above. Dentin disks embedded in paste or gel were incubated at 37 °C under humid conditions for 7 days. After that, dentin disks were flushed by ddH_2_O to remove any remnant paste or gel and the microhardness was measured again. After that, the average microhardness before and after treatment of each group were analyzed and compared.

### 2.12. In Vitro Mineralization

Briefly, 50 mg MCSNs or M-AgTX was compacted to a tablet with a diameter of 13 mm and thickness of less than 1 mm. The tablets were soaked in simulated body fluid (SBF) for 7 days at 37 °C. The formation of mineral crystals on the surface were checked by FE-SEM and EDS analysis, the crystalline phase was examined by Wide-angle X-ray diffraction (XRD) analysis on an X-ray q12 diffractometer (X’Pert PRO, PANalytical, Armelo, The Netherlands) using Cu-Kα radiation at 40 kV and 40 mA, in the 2θ range from 10° to 70° with a scanning speed of 4°/min to determine.

### 2.13. Statistical Analysis

The distribution of data in this study was checked by a skewness-kurtosis test. The data in normal distribution were analyzed using one-way ANOVA with a post hoc Dunnett *t*-test, and for the data violating the normal distribution, non-parametric Mann-Whitney analysis was performed using the SPSS software (IBM, Armonk, NY, USA). Differences for which *p*-values were less than 0.05 were considered statistical significance.

## 3. Results

### 3.1. Characterization of MCSNs and M-AgTX

**Figure 1:**
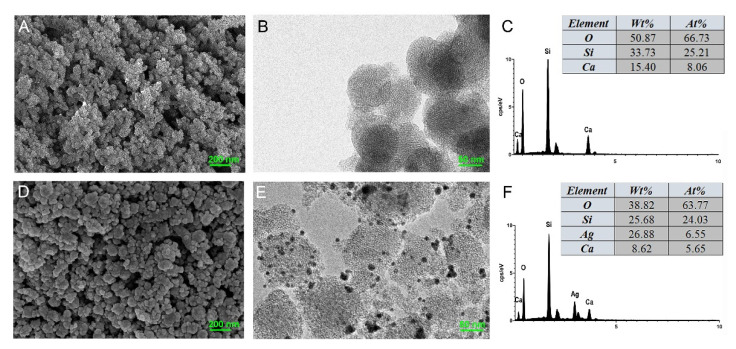
Characterization of MCSNs and M-AgTX. (**A**,**D**) Representative images of MCSNs and M-AgTX by FE-SEM; (**B**,**E**) Representative images of MCSNs and M-AgTX by TEM; (**C**,**F**) Representative images of MCSNs and M-AgTX by EDS.

FE-SEM and TEM revealed that MCSNs and M-AgTX possessed spherical morphology with less than 100 nm diameter (Figure 1A,B,D,E). TEM images showed that the mesoporous structures had well-ordered nanopores and channel structures, and numerous black dots were trapped within the M-AgTX nanoparticles (Figure 1B,E). EDS analysis showed the existence and weight percentage of Ca and Si elements in MCSNs (Figure 1C), and Ag, Ca and Si elements in M-AgTX (Figure 1F).

**Figure 2:**
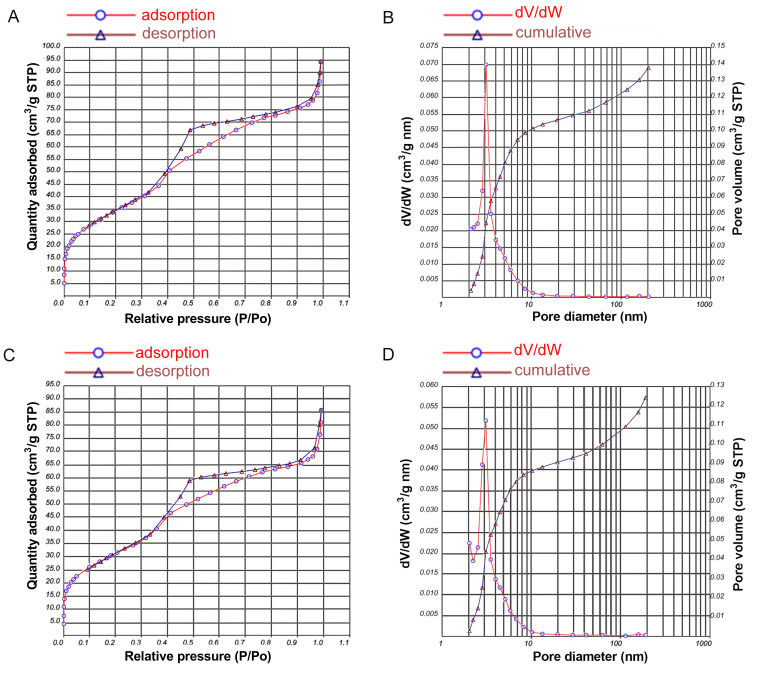
Nitrogen adsorption-desorption isotherm test and pore size distribution of MCSNs and M-AgTX. (**A**,**C**) Nitrogen adsorption-desorption isotherm test of MCSNs and M-AgTX, respectively; (**B**,**D**) Pore size distribution of MCSNs and M-AgTX, respectively.

The result of a nitrogen adsorption–desorption isotherms test is shown in Figure 2, indicating a type IV isotherm with H1-type hysteresis loops, and the pore size of the mesoporous material fell mainly in the 2–6 nm range. The average pore size, pore volume and specific surface area of MCSNs and M-AgTX were presented in Table 1.

**Figure 3:**
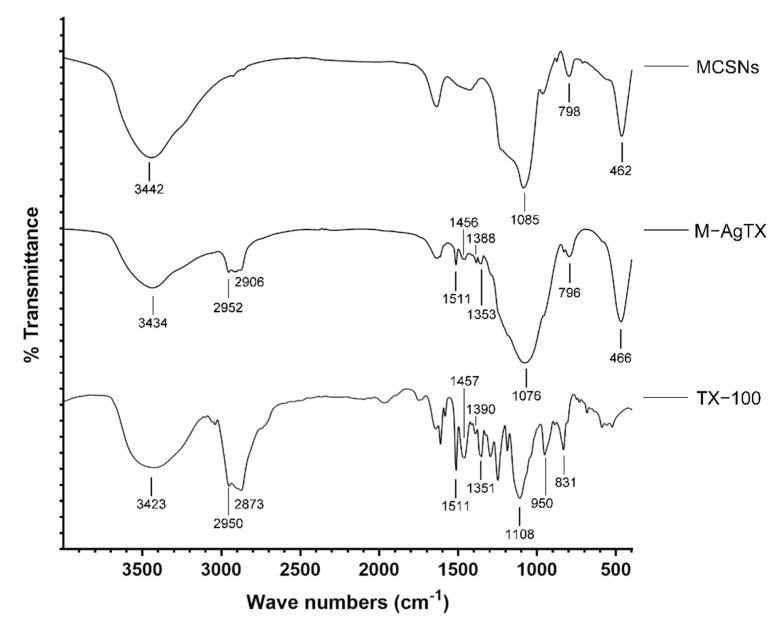
FTIR spectrum of MCSNs, M-AgTX and TX-100.

The FTIR was used to determine the chemical composition (Figure 3). In the spectrum of MCSNs and M-AgTX, the broad band at approximately 3400 cm^−1^ was the specific adsorption peak of hydroxyl groups (−OH); other typical bands were around 1080, 800, and 460 cm^−1^ for Si–O–Si vibration, indicating the existence of silicon skeleton structure in MCSNs and M-AgTX. In the spectrum of TX-100 and M-AgTX, several bands at 1400~1600 cm^−1^ were the specific adsorption peak of benzene skeleton vibration. The bands at approximately 2846~2975 cm^−1^ were the specific adsorption peak of methyl and methylene groups (−CH_3_ and −CH_2_−).

### 3.2. pH Measurement and Release Profile

**Figure 4:**
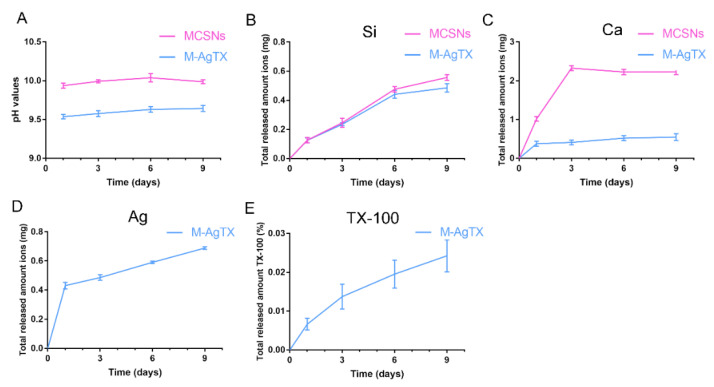
pH and release profile of MCSNs and M-AgTX. (**A**) pH curve of MCSNs and M-AgTX; (**B**,**C**) Total released amount of SiO_3_^2−^, Ca^2+^ of MCSNs and M-AgTX, respectively; (**D**,**E**) Total released amount of Ag^+^ and TX-100 of M-AgTX, respectively.

The pH value of MCSNs was stabilized at around 10, while the value of M-AgTX was always a little lower and stabilized at 9.5 (Figure 4A). ICP-AES test revealed that the release of SiO_3_^2−^ and Ag^+^ were in a sustained increasing manner for over 7 days (Figure 4B,D). While the nanoparticles presented a burst release of Ca^2+^ in the first 3 days, then the curve flattened with a slight increase, and MCSNs release much more Ca^2+^ than M-AgTX (Figure 4C). The results of HPLC showed the amount of TX-100 released showed a stable increase for over 7 days (Figure 4E).

### 3.3. Cytotoxicity Test

**Figure 5:**
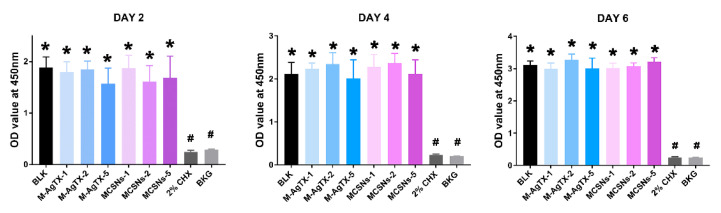
CCK-8 results among groups. (BLK: blank control group; BKG: medium background; M-AgTX-1, M-AgTX-2, M-AgTX-5: 1, 2, 5 mg/mL M-AgTX groups; MCSNs-1, MCSNs-2, MCSNs-5: 1, 2, 5 mg/mL MCSNs groups; *, #: significant difference when compared with 2% CHX group, and BLK group, respectively, *p* < 0.05.).

CCK-8 test in Figure 5 indicated that all nanoparticle groups regardless of concentrations or exposure time exhibited no suppressive effect on the proliferation of MC3T3-E1 cells when compared with the blank control group (*p* > 0.05), whereas the 2% CHX group completely suppressed cell growth at all time points (*p* < 0.05).

### 3.4. MIC and MBC of MCSNs and M-AgTX

MIC and MBC of MCSNs extract were undetectable because of its limited anti-bacterial ability. MIC and MBC of M-AgTX extract were both 16 mg/mL (Table 2).

### 3.5. Anti-Bacterial Effect against Planktonic E. faecalis

**Figure 6:**
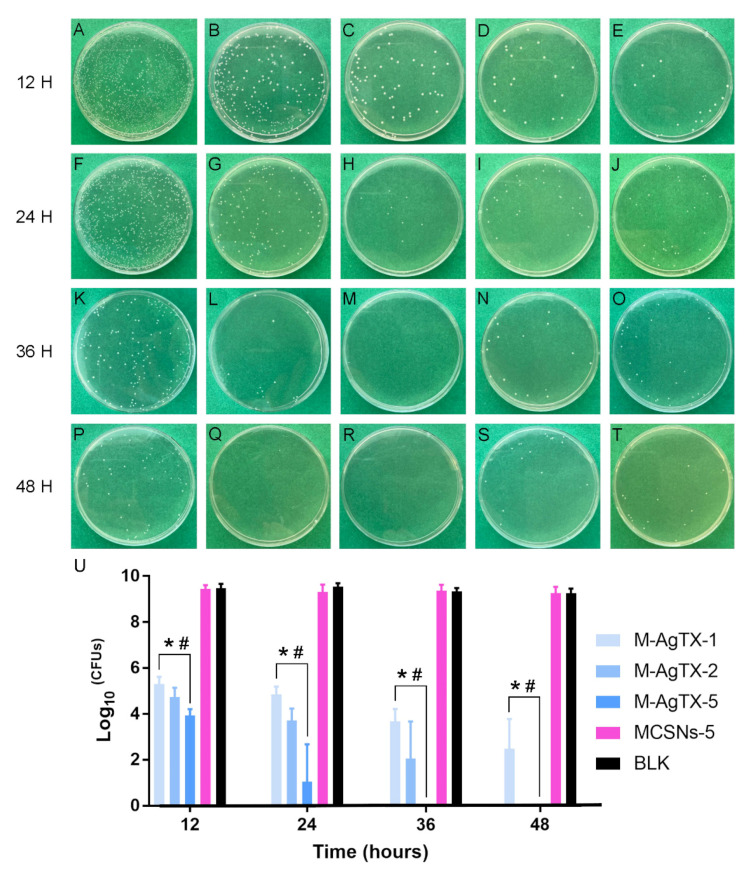
Anti-bacterial effects of different concentrations of materials against planktonic *E. faecalis* at different time intervals. (**A**–**C**,**F**–**H**,**K**–**M**,**P**–**R**) Representative images of CFUs of 1, 2, 5 mg/mL M-AgTX groups at 12, 24, 36, 48 h; (**D**,**I**,**N**,**S**) Representative images of CFUs of 5 mg/mL MCSNs group at 12, 24, 36, 48 h after 10^6^ times dilution; (**E**,**J**,**O**,**T**) Representative images of CFUs of BLK at 12, 24, 36, 48 h after 10^6^ times dilution; (**U**) Log_10_ ^(CFU^^s)^ of groups. (BLK: blank control group; M-AgTX-1, M-AgTX-2, M-AgTX-5: 1, 2, 5 mg/mL M-AgTX groups; MCSNs-5: 5 mg/mL MCSNs group; *, #: significant difference when compared with MCSNs-5 group and BLK group, respectively, *p* < 0.05.).

The anti-bacterial effect of nanoparticles against planktonic *E. faecalis* is shown in Figure 6 and Table 3. After 24-h treatment, 5 mg of M-AgTX could eliminate 10^8^ CFUs bacteria completely in a 1 mL system, and after another 24 h, 2 mg/mL M-AgTX group also achieved the same effect. Although 1 mg/mL M-AgTX group didn’t exhibited a complete bactericidal effect, the survival rate of all M-AgTX groups was significantly higher than the MCSNs and blank control groups (*p* < 0.05). The MCSNs didn’t show significant inhibitory effect on planktonic *E. faecalis* (*p* > 0.05).

### 3.6. Dentinal Tubule Infiltration Ability and Anti-Bacterial Effect on Root Canal Walls Pretreated with M-AgTX

**Figure 7:**
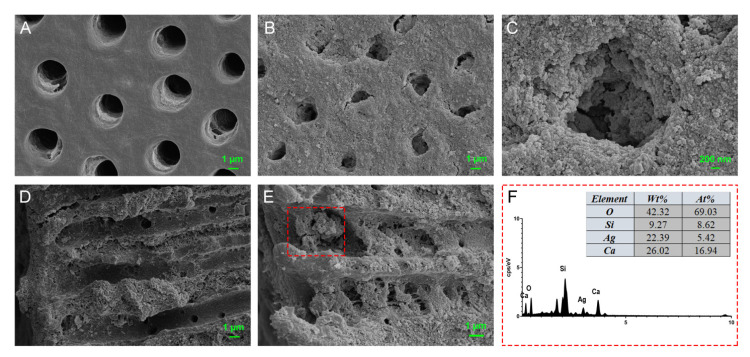
Infiltration of M-AgTX into dentinal tubules. (**A**) Original dentinal tubule openings (**A**) ×5000; (**B**,**C**) Tubule openings treated ultrasonically with M-AgTX (**B**) ×5000, (**C**) ×20,000); (**D**) Original dentinal tubule axial cross sections (**D**) ×5000; (**E**) Tubule axial cross sections after being treated with M-AgTX (**E**) ×7000; (**F**) EDS spectrum of selected square area in (**E**).

In Figure 7A,D, the dentinal tubule openings and axial cross sections were clean before treatments. After ultrasonic activation, M-AgTX nanoparticles were not only observed in tubule openings (Figure 7B,C), but also found in the axial sections of tubules (Figure 7E). The presence of Ag^+^ from the M-AgTX nanoparticles inside the tubules was confirmed by EDS analysis (Figure 7F).

**Figure 8:**
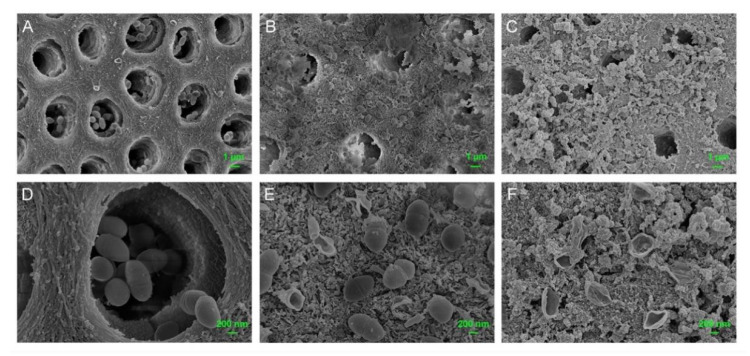
FE-SEM images showing anti-bacterial effects on pretreated dentin slices. (**A**,**D**) *E. faecalis* grown on dentin slice of blank control group (**A**) ×5000, (**D**) ×20,000; (**B**,**E**) *E. faecalis* grown on dentin slice of MCSNs pre-treated group (**B**) ×5000, (**E**) ×20,000; (**C**,**F**) *E. faecalis* grown on dentin slice of M-AgTX pre-treated group (**C**) ×5000, (**F**) ×20,000.

The FE-SEM images further confirmed numerous nanoparticles left on dentin surfaces in both MCSNs group and M-AgTX group even after 7-day immersing treatment (Figure 8B,C).

**Figure 9:**
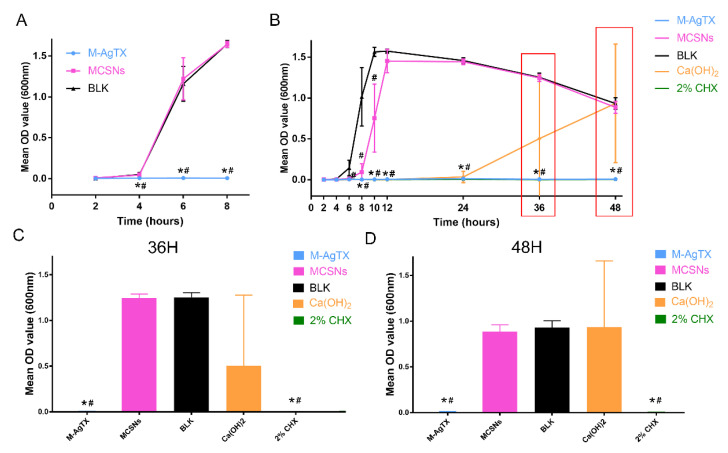
The OD value after the direct soaking of dentin slices from different treatments in fresh BHI media. (**A**) Pretreated dentin slices; (**B**) Dentin slices with 4-week *E. faecalis* biofilm after 7-day medication; (**C**,**D**) Comparisons of OD value at 600 nm at 36 h and 48 h of (**B**). (*,#: significant difference when compared with MSCNs group and BLK group, respectively, *p* < 0.05.).

Besides, there were less bacteria colonizing the dentin surface in the M-AgTX pre-treated group, and the morphology of bacteria in the M-AgTX group changed dramatically with all the cells being shrunk or broken (Figure 8C,F). The OD value measurement revealed that almost no bacteria survive after being treated by M-AgTX as the OD value did not increase during 8 h after immersion in fresh BHI, which was significantly different from both MCSNs and blank control groups (Figure 9A, *p* < 0.05).

### 3.7. Anti-Biofilm Activities of M-AgTX against E. faecalis Mature Biofilm

**Figure 10:**
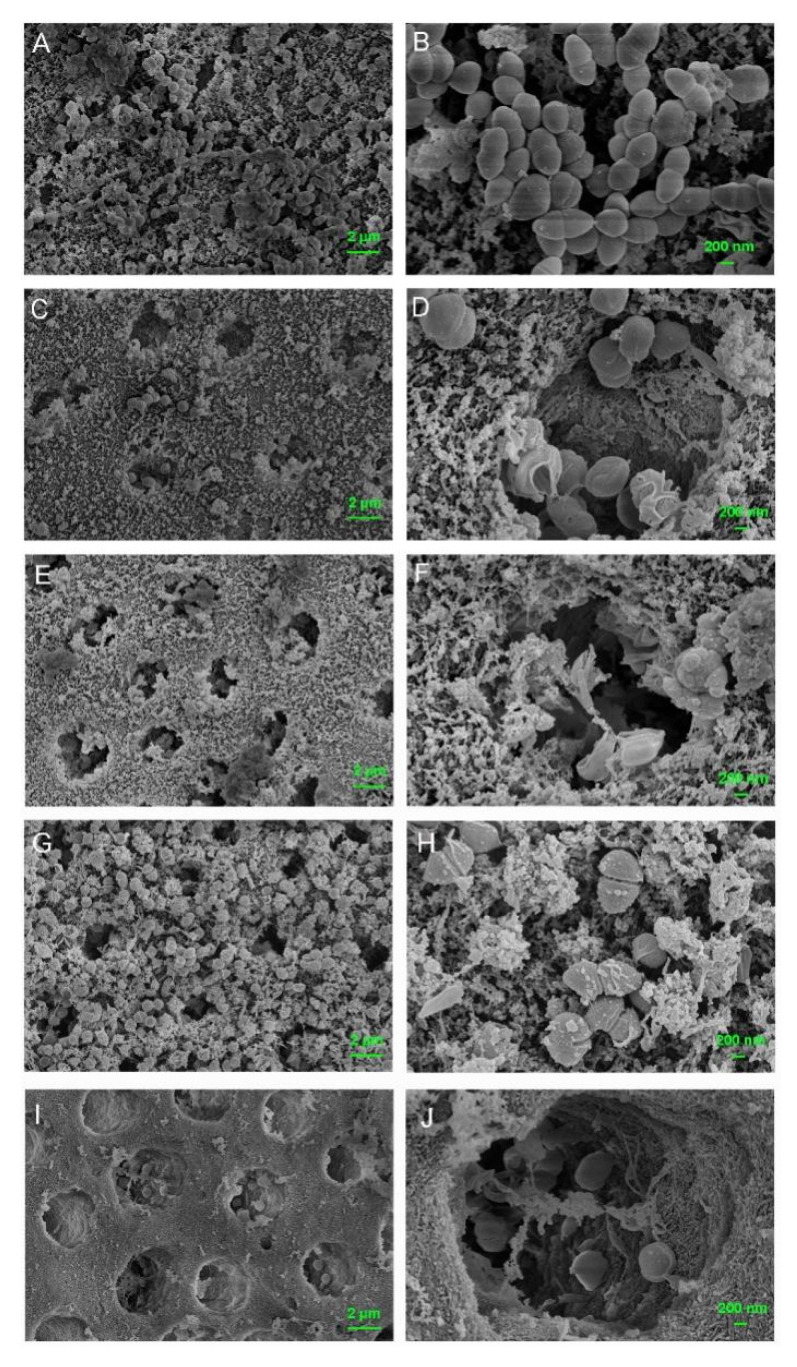
FE-SEM images of 4-week *E. faecalis* biofilm on dentin slices after 7-day medication. (**A**,**B**) biofilm treated with PBS gel (**A**) ×5000, (**B**) ×20,000; (**C**,**D**) biofilm treated with MCSNs paste (**C**) ×5000, (**D**) ×20,000); (**E**,**F**) biofilm treated with M-AgTX paste (**E**) ×5000, (**F**) ×20,000; (**G**,**H**) biofilm treated with 2% CHX gel (**G**) ×5000, (**H**) ×20,000; biofilm treated with Ca(OH)_2_ paste (**I**) ×5000, (**J**) ×20,000).

The OD value revealed that no bacteria survived after being treated with M-AgTX paste or 2% CHX gel groups as the curve didn’t change over time (Figure 9B, *p* < 0.05). The dynamic curve of MSCNs group showed a certain anti-bacterial effect as the plateau stage was reached at around 12 h, which was 2 h later than the BLK group. Besides, in four of six dentin slices treated by Ca(OH)_2_ paste, there were still some bacteria left alive, which resulted in the proliferation of bacteria at 36 or 48 h (Figure 9C,D). The morphology of bacteria treated with MCSNs and M-AgTX paste in Figure 10 were similar to what is seen in Figure 8.

### 3.8. Endocytosis of Nanoparticles by E. faecalis

**Figure 11:**
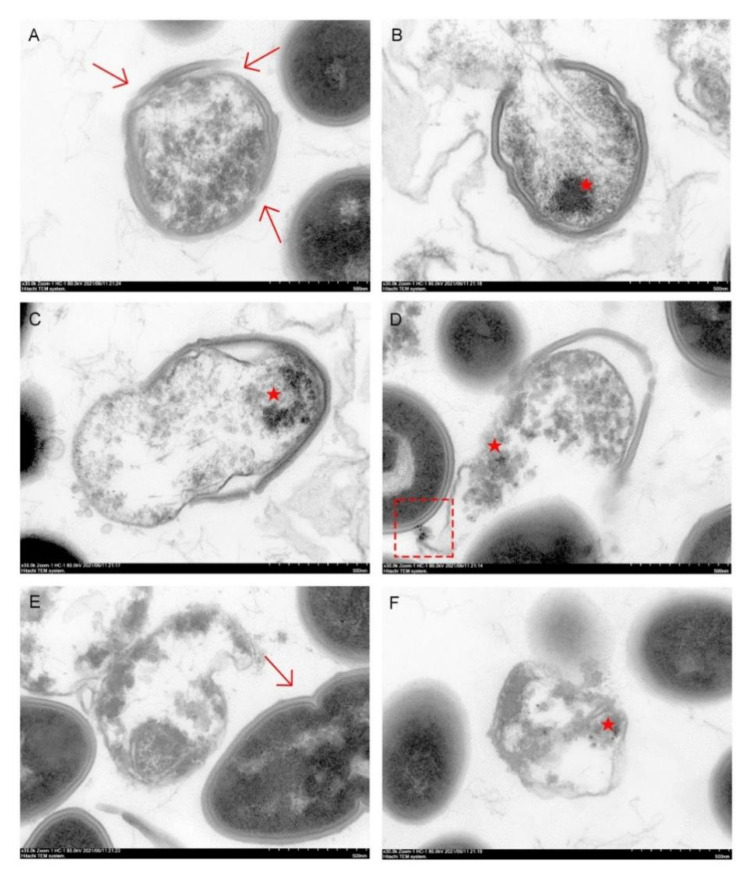
TEM images of endocytosis behavior of *E. faecalis* after being treated with M-AgTX. (**A**–**F**) Different degree of biological membrane defect. (★ indicating the nanoparticles, arrows in (**A**) indicating the cell membrane defect, square frame in (**D**) indicating the M-AgTX outside the cell, and arrow in (**E**) indicating the normal morphology.

Live *E. faecalis* showed typical intact cell membranes in the TEM images (arrow in Figure 11E). After being treated with M-AgTX, *E. faecalis* showed different degrees of destruction of the cell wall and membranes, and the M-AgTX could be seen inside the bacteria (Figure 11A–F). Some nanoparticles could also be seen aggregated and attached to the *E. faecalis* cell wall, as shown in the Figure 11D.

### 3.9. Microhardness Test

**Figure 12:**
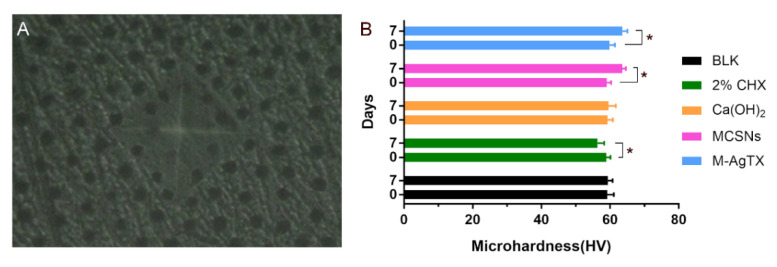
Dentin microhardness measurement. (**A**) Representative image of dentin slices in microhardness measurement (×40); (**B**) Comparison of microhardness among groups. (*: *p* < 0.05).

As shown in Figure 12 B, there was no statistical difference in the change of microhardness for BLK and Ca(OH)_2_ groups (*p* > 0.05), the microhardness of MCSNs and M-AgTX groups increased after treatment (*p* < 0.05), while 2% CHX group displayed a slight reduction (*p* < 0.05).

### 3.10. In Vitro Mineralization

**Figure 13:**
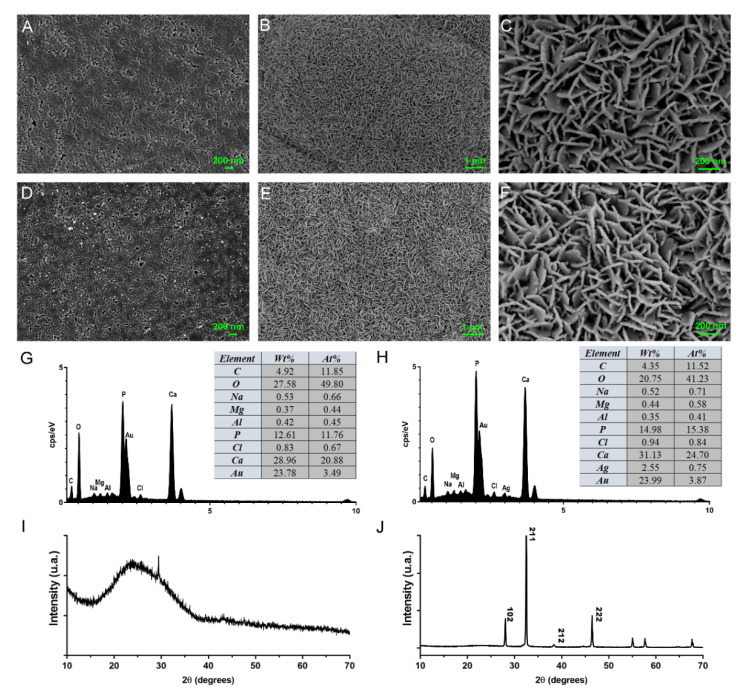
In vitro mineralization of MCSNs and M-AgTX. (**A**,**D**) FE-SEM image of original MCSNs and M-AgTX tablets surfaces, respectively; (**B**,**C**) FE-SEM image of MCSNs tablet surface after being soaked in SBF for 7 days (**B**) ×10,000, (**C**) ×50,000); (**E**,**F**) FE-SEM image of MCSNs tablet surface after being soaked in SBF for 7 days (**E**) ×10,000, (**F**) ×50,000; (**G**,**H**) EDS spectrum of crystals formed on MCSNs and M-AgTX tablets surfaces after being soaked in SBF for 7 days, respectively; (**I**,**J**) Wide-angle XRD patterns of M-AgTX before and after being soaked in SBF for 7 days.

FE-SEM images revealed that after the 7-day immersion in SBF, massive apatite crystals could be induced on the surface of MCSNs and M-AgTX tablets (Figure 13B,E). EDS analysis revealed that Ca, P elements in these newly formed crystals, and the Ca/P ratio was in the range of 2.08–2.29 (Figure 13G,H). For wide-angle XRD, non-crystalline scattering is observed for M-AgTX with no treatment between 10° and 40°, implying the material is in an amorphous state; and the result of M-AgTX after being soaked in SBF for 7 days presented a main peak at around 2θ = 32° (211) and other typical peaks, which are characteristic for the standard diffraction peaks of the hydroxyapatite phase (JCPDS 09-0432) (Figure 13I,J) [28].

## 4. Discussion

In this study, a M-AgTX biocomposite was successfully fabricated by the adsorption method, which was confirmed by FTIR, EDS, TEM, nitrogen-adsorption isotherms and release profiles. These characteristics are consistent with previous studies on calcium silicate-based materials [15,29,30]. The results of FTIR revealed that M-AgTX preserved the specific adsorption peak of benzene skeleton and silicon skeleton structure, suggesting the incorporation of TX-100 within M-AgTX [28,30,31]. Although the chemical mechanism behind this combination is still undetermined, the active sites of Ca-OH and Si-OH groups of MCSNs and −CH_2_ groups of TX-100 could very much likely bind together through a covalent binding process under a heated-stirring condition [13,32]. When considering the EDS and pH curve, the Ca^2+^ cations on the surface of M-AgTX might serve as the inherent active site to effectively adsorb TX-100 with -OH groups [17]. The above speculation could also possibly explain why M-AgTX has a relatively lower pH value and less Ca^2+^ released amount than MCSNs.

In addition, the co-release of Ag^+^ and TX-100 in M-AgTX could further enhance the antibacterial ability. In the anti-bacterial effect against planktonic *E. faecalis* test, low-dose M-AgTX could eliminate the bacteria in a concentration-dependent as well as time-dependent manner. Besides, studies have shown that bacteria biofilm are up to 1000 times more resistant to antibodies, phagocytosis and antibiotics than their free-floating or ‘planktonic’ counterparts [33]. Therefore, the complete destruction of biofilms is of great significance in root canal disinfection. The result from measuring OD value in Figure 9B revealed that no *E. faecalis* survived in the 4-week biofilm after treated by M-AgTX paste as the 2% CHX gel, while there were still some bacteria left in Ca(OH)_2_ paste treated group, which is consistent with previous studies [8,34,35]. As shown in Figure 8, Figure 10 and Figure 11, the morphology of bacteria treated by M-AgTX either before or after infection showed different degrees of cell breakdown, from perforation to rupture, and M-AgTX particles were clearly observed inside the bacteria cells. TX-100 was reported to enhance the susceptibility of the bacteria to drugs by increasing the penetrability of cell membrane [36,37,38]. The results of this study confirmed the role of TX-100 in increasing the permeability of bacteria cells and promoting the infiltration of M-AgTX. Through this mechanism, TX-100 could possibly decrease both alkaline and Ag^+^-resistance of *E. faecalis*, and then in turn enhance the antibacterial effect of silver and hydroxide ions in M-AgTX against *E. faecalis.*

Moreover, the morphology of bacteria treated by 2% CHX gel in Figure 8; Figure 10 remained intact. CHX can fix but not dissolve the bacteria, and the surface layer of the biofilm treated with CHX would block further penetration of CHX into the deeper area [39]. Sharmila et al. [40] found that the penetration depth of 2% CHX to the mature biofilm of *E. faecalis* was only 200 µm. Many commercial anti-bacterial agents such as Q-Mix, MTAD, REDTA et al. use surfactants to help reduce the surface tension and increase the wettability of the solutions and achieve the effect of disintegrating biofilms [41]. Therefore, given the significant inhibition of biofilm after M-AgTX treatment, M-AgTX might penetrate into the deep layer of the biofilm and disrupt it with the help of TX-100.

The MIC and MBC of M-AgTX were both 16 mg/mL, which were different from the result of anti-planktonic *E. faecalis* test. Fan et al. [13] ever found that ionic surfactant CHX had a higher affinity for mesoporous materials and could be re-adsorbed on the surface of mesoporous materials after release. It might be similarly surmised that nonionic surfactant TX-100 might also re-absorbed by the M-AgTX after release, hence the direct contact between M-AgTX and bacteria may be beneficial for a better anti-bacterial activity.

Apart from anti-biofilm activity, M-AgTX nanoparticles could also be driven into the dentinal tubules by ultrasonic activation and fuse with the dentin, turning the tubules into a potential anti-bacterial agent reserve and contributing to the continuous anti-bacterial effect against re-infection. In addition, the microhardness of the treated dentin increased and may help prevent root fracture after root canal medication. In vitro mineralization test revealed that the hydroxyapatite-like crystallization was confirmed by XRD. This rapid mineralization is partly due to the stable release of Ca^2+^ and SiO_3_^2−^, which could further favor the biomineralization and hard tissue regeneration [17]. What’s more, compared with 2% CHX, M-AgTX showed no negative effect on the proliferation of pre-osteoblast cells. Considering these features, M-AgTX might also be developed into a new bone defect filling material.

## 5. Conclusions

M-AgTX showed sustained release profile, low cytotoxicity, infiltration ability into dentinal tubules, enhanced anti-bacterial and mineralization features. TEM confirmed the destruction of cell membrane integrity and Ag^+^ infiltration into bacteria by M-AgTX. Besides, dentin slices medicated with M-AgTX nanoparticles displayed an increased microhardness. These findings might indicate that M-AgTX could possibly be developed into a new multifunctional intracanal medication or bone defect filling material for infected bone defects. Further studies to investigate the anti-multi-species bacteria ability as well as the in vivo application of M-AgTX are necessary before it could be translated into clinical practices.

## Figures and Tables

**Table 1:** The surface area (S_BET_), pore volume (V_P_), and mean pore size (D_P_) of MCSNs and M-AgTX.

Groups	S_BET_ (m^2^ g^−1^)	V_P_ (cm^3^ g^−1^)	D_P_ (nm)
MCSNs	120.27	0.15	4.66
M-AgTX	109.32	0.13	4.65

**Table 2:** MIC and MBC values of MCSNs and M-AgTX extracts against *E. faecalis*.

Groups	MIC (mg/mL)	MBC (mg/mL)
MCSNs	-	-
M-AgTX	16	16

**Table 3:** Survival rate (%) of MCSNs and M-AgTX against planktonic *E. faecalis*.

Times/Groups	M-AgTX-1	M-AgTX-2	M-AgTX-5	MCSNs-5	BLK
12 h	56.01% ± 4.74% *^,#^	49.96% ± 6.18% *^,#^	41.44% ± 1.90% *^,#^	99.64% ± 1.24%	1
24 h	50.91% ± 4.68% *^,#^	39.03% ± 4.30% *^,#^	11.13% ± 21.54% *^,#^	97.64% ± 4.32%	1
36 h	39.38% ± 10.83% *^,#^	22.16% ± 23.80% *^,#^	0.00% ± 0.00% *^,#^	100.51% ± 4.61%	1
48 h	26.91% ± 8.80% *^,#^	0.00% ± 0.00% *^,#^	0.00% ± 0.00% *^,#^	100.02% ± 0.96%	1

M-AgTX-1, M-AgTX-2, M-AgTX-5: 1, 2, 5 mg/mL M-AgTX groups; MCSNs-5: 5 mg/mL MCSNs group; *,^#^: significant difference when compared with MCSNs-5 group, and BLK group, respectively, *p* < 0.05.

## Data Availability

All data generated or analyzed during this study are included in this published article.
